# Evolution du système national d’information sanitaire de la république démocratique du Congo entre 2009 et 2015

**DOI:** 10.11604/pamj.2017.28.225.13894

**Published:** 2017-11-14

**Authors:** Saleh Muhemedi, Yakim Kabangu, Faustin Mpeli, Salomon Salumu, Philippe Kabeya, Emile Okitolonda

**Affiliations:** 1 Ecole de Santé Publique de l’Université de Kinshasa, Kinshasa, République Démocratique du Congo; 2 Direction du SNIS, Secrétariat Général, Ministère de la Santé Publique, RD Congo; 3 Cellule de Centralisation des données, Secrétariat Général, Ministère de la Santé Publique, Kinshasa, RD Congo

**Keywords:** Outil HMN, SNIS, RD Congo, HMN tool, NHIS, DR Congo

## Abstract

**Introduction:**

Lancé en 1987, le Système national d'information sanitaire (SNIS) de la République Démocratique du Congo (DR Congo) a été évalué en 2009 et 2015 moyennant l'outil HMN (Health metrics network). L'objectif de cette étude était d'estimer les progrès réalisés entre ces deux évaluations.

**Méthodes:**

Il s'agissait d'une analyse des données secondaires des évaluations du SNIS, qui a consisté à comparer le degré de satisfaction par rapport aux six composantes de cet outil entre 2009 et 2015, à savoir; les ressources, les indicateurs, les sources de données, la gestion des données, les produits de l'information, la diffusion et l'utilisation des données.

**Résultats:**

Entre 2009 et 2015, le degré de satisfaction des répondants a évolués de la manière suivante: 49% contre 61% pour les ressources; 73% vs 88% pour les indicateurs; 52% vs 61% pour les sources de données; 41% vs 45% pour la gestion des données; 74% contre 77% pour les produits de l'information et enfin 51% vs 51% pour la diffusion et l'utilisation des données. Dans l'ensemble, le score moyen est passé de 59% à 64% avec une mention « satisfaisante ».

**Conclusion:**

Notre étude a montré que le SNIS de la RD Congo n'a pas significativement évolué entre 2009 et 2015, et n'était pas en mesure de fournir en temps réel l'information sanitaire fiable pour la prise de décision et la planification des programmes de santé.

## Abstract (en)

**Introduction:**

Launched in 1987, the National Health Information System (NHIS) in the Democratic Republic of the Congo (DR Congo) was evaluated in 2009 and 2015 using the HMN (Health Metrics Network) assessment tool. This study aimed to estimate the progress made between these two evaluations.

**Methods:**

We performed an analysis of the secondary data from the evaluations of the NHIS, which was based on the comparison of the satisfaction level between the six components of this tool between 2009 and 2015, namely: resources, indicators, data sources, data management, information products, dissemination and use of data.

**Results:**

Between 2009 and 2015, respondents' satisfaction level changed as follow: resouces 49% vs 61%; indicators 73% vs 88%; data sources 52% vs 61%; data management 41% vs 45%; information products 74% vs 77%; dissemination and use of data 51% vs 51%. In general the average score increased from 59% to 64% with "satisfactory" mark.

**Conclusion:**

Our study shows that the NHIS in the DR Congo has not changed significantly between 2009 and 2015 and that it couldn't provide real-time reliable health information for decision-making and health program planning.

## Introduction

Un bon Système d'Information Sanitaire (SIS) est une base indéniable dans la planification, la mise en œuvre, le suivi et l'évaluation des programmes de santé [1-4]. En effet, le SIS est un des six piliers fondamentaux du système de santé dont le rôle est de produire, d'analyser et de diffuser à temps les données fiables et opportunes devant servir à la prise de décisions en vue d'améliorer l'état de santé de la population [5-8]. C'est ainsi que les performances d'un tel système devraient être mesurées non seulement sur base de la qualité de l'information produite mais aussi sur les preuves de son usage continu pour l'amélioration des programmes de santé et de la situation sanitaire d'un pays [1, 8]. En vue de mesurer ses performances et d'assurer la comparabilité des résultats d'un pays à l'autre, l'Organisation Mondiale de la Santé (OMS) et ses partenaires ont créé à la fois une plate-forme dénommée « Réseau de Métrologie sanitaire (RMS) » ou « Heath Metrics Network (HMN) » en anglais et un outil mieux connu sous l'appellation « outil HMN » [9, 10]. Ce réseau avait comme objectifs: (i) créer un cadre harmonisé pour le développement des systèmes d'information sanitaire nationaux obéissant à certaines normes; (ii) renforcer ces systèmes en offrant un appui technique et financier; (iii) établir des politiques, mécanismes et mesures incitatives propres à améliorer l'accès des parties locales, régionales et mondiales intéressées à l'information sanitaire et l'usage qu'elles en font [11] Depuis 2007, certains pays africains ont pu utiliser l'outil HMN pour évaluer leurs systèmes d'information sanitaire; notamment le Burkina Faso [12], le Burundi [13], la Cote d'ivoire [14], pour ne citer que ceux-là. D'une manière générale, la revue de la littérature a montré une différence notable entre ces pays par rapport au niveau de réalisation de ces six composantes, qui pour certains chercheurs, est expliquée par des facteurs tels que l'organisation mis en mise, la technologie utilisée, la disponibilité des ressources, la demande des usagers du système d'information, le comportement du personnel chargé de la gestion des données (de la collecte à l'utilisation de l'information générée), la gouvernance [15-17]. La République Démocratique du Congo (RD Congo) a participé au lancement du RMS dans la région francophone en 2005 à Dakar et à l'atelier de formation sur l'outil HMN à Cotonou en 2007. En 2009 et en 2015, elle a procédé à deux évaluations de son SNIS en utilisant l'outil HMN adapté, dont la comparaison des résultats fait l'objet de cette étude.

## Méthodes

Il s'agissait d'une analyse des donnés secondaires qui a consisté à comparer les résultats des évaluations du SNIS de la RD Congo effectuées en 2009 et en 2015. Cette analyse a porté sur le niveau de satisfaction des utilisateurs du SNIS moyennant l'outil HMN. Cette comparaison a concerné les six composantes de cet outil et ses attributs ou sous composantes ci-dessous: Pour les ressources, il s'agissait d'apprécier l'environnement politique, réglementaire et financier qui a été mis en place ainsi que des infrastructures et ressources disponibles conditionnant un fonctionnement optimal du SNIS. Concernant les indicateurs, l'appréciation a porté sur les indicateurs de base, axés principalement sur la prévention des risques, la prise en charge des maladies, la promotion des conditions propices pour la santé. Pour ce qui est de sources de données, six principales sources ont été identifiées, à savoir: le recensement de la population, les statistiques de l'Etat-civil, les enquêtes dans la population, les rapports sanitaires et les registres des malades, les registres et les rapports des services de santé ainsi que les registres administratifs. En ce qui concerne la gestion de données, la comparaison a porté sur l'examen des processus optimaux de collecte, de partage, de stockage, de flux de données et des boucles de rétroaction. Quant aux produits de l'information: il s'agissait d'examiner les variables de la qualité des données disponibles telles que la transparence, la désagrégation, la représentativité, la cohérence, la périodicité, la ponctualité/actualité et la méthode de collecte des données. Concernant la dissémination et l'utilisation de l'information sanitaire, l'appréciation s'est focalisée sur la portée de la diffusion de l'information et l'usage que les parties prenantes du SNIS en font pour la prise des décisions.

Cela incluait l'élaboration des politiques, le plaidoyer, la planification, la mise en œuvre des interventions ainsi que le suivi et l'évaluation. Pour chaque évaluation du SNIS de la RD Congo, un atelier de remplissage de l'outil a été organisé, atelier au cours duquel six groupes thématiques comprenant les utilisateurs du système ont été constitués selon les composantes de cet outil. Chaque groupe comprenait au moins dix évaluateurs qui individuellement avaient attribué un score entre 0 et 3 aux items de leur composante et un score moyen par item a été calculé. Au total 167 items ont été cotés pour les six groupes. L'outil lui-même est un fichier Excel programmé produisant de façon automatique des résultats de l'évaluation en pourcentage, regroupé en quatre mentions ou quartiles définies sur une échelle ordinale et traduisant le degré de satisfaction des répondants. Il s'agit de: La mention « non satisfaisante du tout », dont le score est ≤ 25%, soit le premier quartile; la mention « présente, mais non satisfaisante », se situe entre 26% et 50% et correspond au deuxième quartile; la mention « satisfaisante », entre 51% et 75% représentant le troisième quartile et la mention « très satisfaisante », entre 76% et 100% et correspond au quatrième quartile. Comme à l'issue des évaluations du SNIS RD Congo, les résultats de cette étude ont été présentés sous forme des diagrammes en barre. L'interprétation des résultats contenus dans les rapports de ces évaluations ont permis d'enrichir la discussion de la présente étude.

**Considérations éthiques:** Etant donné que notre étude a consisté à une analyse des données secondaires relatives aux évaluations du SNIS 2009 et 2015 dont les résultats ont été déjà diffusés, et compte tenu du caractère anonyme et impersonnel de ces données, nous n’avons pas trouvé indispensable de recourir à l’approbation du comité d’éthique.

## Résultats

**Figure 1:**
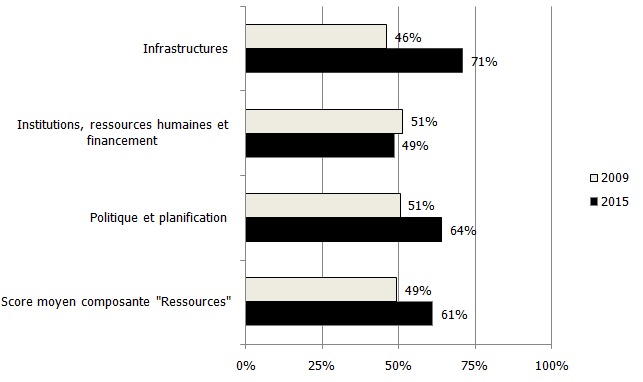
Niveau de satisfaction de la composante « ressource » en 2009 et 2015

**Composante « ressources »**: Les résultats de cette composante montre qu'il y a eu une nette progression en 2015 par rapport en 2009 avec un score moyen qui est passé de 49% à 61%. Ces résultats correspondaient au passage de la mention « présente, mais non satisfaisante » à la mention « satisfaisante ». L'examen de cette composante a montré que les sous-composantes infrastructures, politiques et planification ont progressé, passant respectivement de 46% à 71% et de 49% à 61%. Par contre, la sous composante « ressources et financement » a légèrement diminué en 2015 par rapport en 2009, passant de 51% à 49% (Figure 1).

**Composante « indicateurs »**: Nous avons noté une progression significative de la composante « Indicateurs » en 2015 par rapport à l'année 2009 avec un score moyen qui est passé de 73% à 88%, soit le passage de la mention «satisfaisante » à la mention « très satisfaisante ».

**Figure 2:**
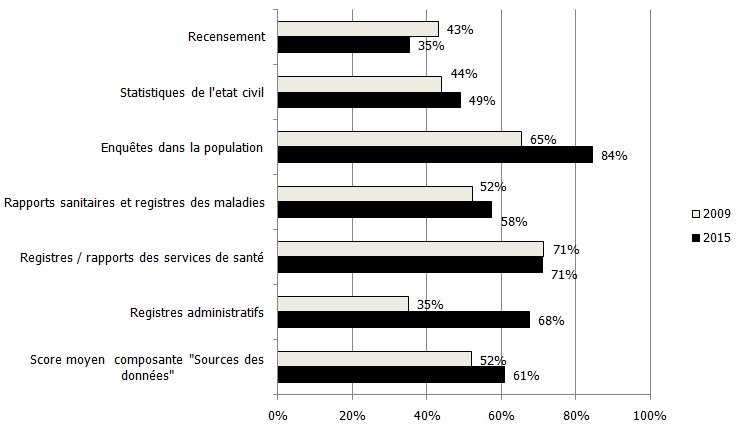
Niveau de satisfaction de la composante « sources des données » en 2009 et 2015

**Composante « sources des données »**: Par rapport en 2009, la composante connu une progression en 2015, uniquement en termes de pourcentage, avec un score moyen qui est passé de 52% à 61%. Cependant, la mention n'a pas changée. Elle est restée « satisfaisante ». L'examen des sous- composantes a montré que les sous-composantes « enquêtes dans la population » et « registres administratifs ont augmenté d'un quartile, passant respectivement de 65% à 84% et de 35% à 68%. En revanche, la sous composante « recensement » a régressé, passant de 45% en 2009 à 35% en 2015. Il faut noter cependant que la sous-composante « registres/ rapports des services de santé » est restée stationnaire avec 71% lors de ce deux évaluations (Figure 2).

**Composante « gestion des données »**: Pour la composante « Gestion des données » le score moyen s'est légèrement amélioré passant de 41% en 2009 à 45% en 2015. Toutefois, la mention est restée inchangée, c'est à dire «présente, mais non satisfaisante ».

**Figure 3:**
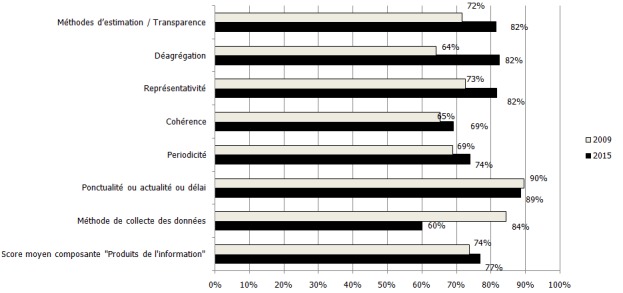
Niveau de satisfaction de la composante « produits de l’information » en 2009 et 2015

**Composante « produits de l'information »**: Par rapport à cette composante, il y a eu dans l'ensemble une progression du score moyen en 2015 par rapport en 2009 avec une mention « satisfaisante » qui est passée à la mention « Très satisfaisante ». Cependant les sous-composantes: « méthode de collecte des données » et « ponctualité/actualité/délai » ont régressé, passant respectivement de 84% en 2009 à 60% en 2015 et de 90% à 89%. En opposé, la progression était notable dans la sous-composante « représentativité » qui a avancée d'un quartile. (73% en 2009 à 82%) (Figure 3).

**Figure 4:**
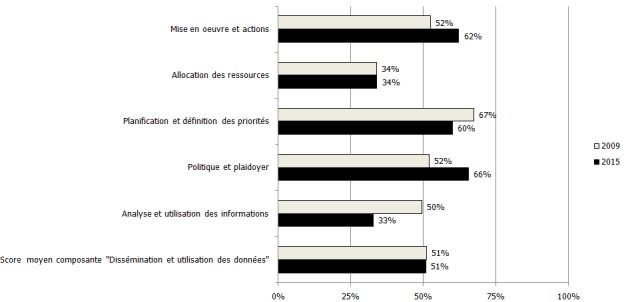
Niveau de satisfaction de la composantes « dissémination et utilisation des données » en 2009 et 2015

**Composante « dissémination et utilisation des données »**: Les résultats obtenus pour cette composante ont montré qu'il y a eu une stagnation du score moyen à 51% en 2009 et en 2015 donnant lieu à une mention « satisfaisante ». Quoiqu'il y ait eu des efforts en matière de plaidoyer et de politique, nous avons constaté cependant une régression dans l'analyse et l'utilisation de l'information (Figure 4).

**Figure 5:**
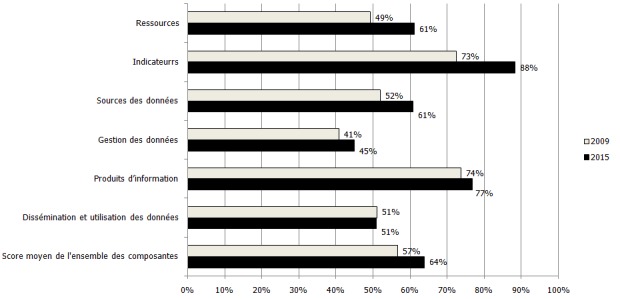
Niveau de satisfaction de l’ensemble des composantes en 2009 et 2015

**Ensemble des composantes** : Dans l'ensemble, le score moyen est passé de 57% en 2009 à 64% en 2015, mais la mention est restée inchangée, c'est-à-dire « satisfaisante » à l'issue de ces deux évaluations (Figure 5).

## Discussion

Entre 2009 et 2015, les résultats de notre étude ont montré que les ressources du SNIS ont progressé, passant de la mention « présente, mais non satisfaisante » à la mention « satisfaisante ». Cela est en partie dû aux efforts fournis par le Ministère de la Santé de la RD Congo dans l'élaboration de politique et la mise en place d'un cadre qui régit la gestion du SNIS afin d'améliorer le processus décisionnel et à l'accroissement du nombre des formations sanitaires tant publiques que privés qui produisent l'information sanitaire. En outre, l'extension du réseau de téléphonie cellulaire et de l'Internet a tant soit peu contribué à l'amélioration de la transmission des données sanitaires comme évoqué dans certaines études [18-21]. Au-delà de ces quelques progrès enregistrés dans cette composante, les deux évaluations du SNIS ont toutes relevé un véritable problème de coordination des activités du SNIS à tous les niveaux de la pyramide comme ce fut le cas au Botswana en 2015 [11] et tel que décrit par Lippeveld T et al [22]. Parmi les problèmes épinglés en 2009 et qui n'ont pas trouvés satisfaction en 2015, figurent l'insuffisance de ressources matérielles (imprimés, équipements et leur maintenance) et humaines qualifiées comme rapporté par Verberk et al en 2004 [23]. Par ailleurs, les dépenses de l'état congolais consacrées au secteur de santé ont été insignifiantes, de l'ordre de moins de trois dollars américains par habitant et par an [24]. Pour les quelques ressources financières octroyées à ce secteur, le SNIS n'est pas été une priorité. Pour ce qui concerne les indicateurs, il y a eu progression de la composante « Indicateurs » entre 2009 et 2015 avec une mention qui est passée de «satisfaisante » à « très satisfaisante ». Au total, 220 indicateurs ont été collectés en routine et 30 collectés par les enquêtes. En 2015, les formations sanitaires ont mieux compris leur formulation et la façon de les collecter qu'en 2009. Les résultats de ces deux évaluations ont aussi montré que les partenaires techniques et financiers ont créé une demande supplémentaire d'indicateurs dont ils avaient besoin. En outre, certains indicateurs essentiels de différents programmes spécialisés du Ministère de la Santé n'ont pas été suffisamment intégrés dans la composante du SNIS relative aux soins de santé primaires [25, 26]. Pour les sources de données, le dernier recensement scientifique de la population et de l'habitat a été organisé en 1984 par l'INS (Institut National des Statistiques) et a rapporté un taux annuel de croissance de la population autour de 3%. A ce jour, ce taux est encore utilisé pour estimer la taille de la population nationale [27]. Cette situation continue à poser un réel problème dans la planification des interventions et la mesure des plusieurs indicateurs. Comme autre sources des données, figure le service de l'Etat Civil dont les données étaient insuffisantes et moins disponibles avec une couverture de 45% en milieu urbain [25].

Ces données n'étaient accessibles qu'à la demande, car non diffusées. A propos des décès par exemple, les données n'ont été enregistrées que dans les hôpitaux et dans quelques cimetières. Leurs causes n'ont été mentionnées que sur les certificats de décès délivrés par certains hôpitaux. Quant aux enquêtes nationales dans la population, quelques-unes ont été organisées entre 2009 et 2015. Nous citerons l'enquête par grappes à indicateurs multiples (MICS 3) organisée en 2010 [28], l'Enquête Démographique et de Santé (EDS) menée en 2013 [29] et l'enquête nationale sur l'analyse globale de la sécurité alimentaire et la vulnérabilité conduite en 2005 [30]. Par rapport à la composante« gestion des données », les résultats de notre étude ont montré un statuquo entre 2009 et 2015 avec une mention « présente, mais non satisfaisante ». Bien que le Ministère de la Santé ait élaboré un cadre normatif SNIS [2, 3] pour l'alignement, beaucoup d'effort restent encore à déployer pour sa mise en œuvre. Certains intervenants, bien qu'associés dans ce processus, ont continué à mettre en place des outils parallèles de collecte des données sur terrain en fonction de leurs besoins, comme ce fut le cas au Malawi en 2005 [21]. En outre, malgré l'implantation du logiciel DHIS2, seules 284 Zones de santé sur les 516 existantes en ont bénéficié [25]. Son hébergement dans le site Web du SNIS a tout de même facilité le partage des données et rentabilisé leur stockage. Entre temps, les imprimés ont restés les seules outils de collecte et de compilation des données au niveau périphérique (centres de santé et hôpitaux généraux de référence). Par ailleurs, les rapports de ces évaluations ont souligné la faiblesse de la retro-information du niveau central vers le niveau périphérique. Quant aux produits de l'information, la qualité des indicateurs sélectionnés, leur ponctualité ou actualité ainsi que la transparence des méthodes d'estimation ont été améliorés en 2015 par rapport en 2009 suite à l'intensification des formations sur la gestion de l'information sanitaire à travers le pays, situation similaire à celle du Botswana en 2015 [11]. Il en est de même de la désagrégation des données qui a permis de répondre aux besoins des utilisateurs et améliorer la traçabilité. Cependant, la performance de la ponctualité ou actualité qui était de 90% en 2009 et de 89% en 2015 est en contradiction avec la faiblesse de la promptitude du SNIS de RD Congo évoquées dans ces mêmes rapports d'évaluation et dans certaines études menées en RD Congo [23]. Pour ce qui est de la dissémination et l'utilisation des données, les évaluations du SNIS ont montré une sous utilisation de l'information sanitaire en général et de nouvelles technologies de l'information en particulier nonobstant les bénéfices qu'on pourrait en tirer [17, 18]. De plus, malgré la production des annuaires et des bulletins, leurs diffusions ont été tardives. Pour ce qui est de l'utilisation des données, les résultats de notre étude ont montré qu'elle demeure un des problèmes majeurs, tant dans la planification que dans la mise en œuvre et le suivi-évaluation du SNIS en RD Congo à tous les niveaux de la pyramide sanitaire, à l'instar de certains pays africains sub-sahariens [2, 21].

## Conclusion

Notre étude portant sur l'évolution du système d'information sanitaire de la République Démocratique du Congo entre 2009 et 2015 a montré que les efforts ont été fournis en termes d'amélioration des ressources, de compréhension des indicateurs et de qualité des données. Néanmoins, ils n'ont pas été suffisants pour améliorer la mention obtenue en 2009 qui est restée la même qu'en 2015, c'est dire « satisfaisante », malgré une amélioration du score moyen des composante qui est passé de 57% à 64%. Des efforts supplémentaires sont nécessaires pour étendre la couverture du DHIS2 dans toute la R D Congo et fournir en temps réel l'information sanitaire fiable qui devrait être diffusée et utilisée pour améliorer la santé de la population congolaise.

### Etat des connaissances actuelle sur le sujet

- Un bon système d'information sanitaire permet la prise des décisions pour l' amélioration de l'état de santé d'une population;
- Un bon fonctionnement d'un système de santé requiert des politiques et directives, des ressources humaines, matérielles (y compris technologiques) et financières et un montage organisationnel efficient;
- L'information produite par le SNIS de la RD Congo est sous utilisée.

### Contribution de notre étude à la connaissance

- Entre 2009 et 2015, notre étude a permis de montrer que le SNIS de la de la RD Congo n'a pas beaucoup progresse malgré quelques efforts entrepris;
- Plus de trois quarts des 316 zones de santé pouvaient utiliser le DHIS2;
- En République Démocratique du Congo, les données continuent à être collectées manuellement et en version papier au niveau des centres de Santé et des Hôpitaux Généraux de Référence.

## Conflits d’intérêts

Les auteurs ne déclarent aucun conflit d'intérêts.
